# To Relieve Morbid Thirst for Alcoholic Drinks

**Published:** 1877-08

**Authors:** 


### EXCERPTA.

---

To Relieve Morbid Thirst for Aocoholic Drink.—S. B. Merkel, M.D., of Philadelphia, writes to the *Journal of Materia Medica* as follows:

"A tonic and stimulant which partially supplies the place of the accustomed liquor, and prevents the absolute mental and physical prostration that follows a sudden breaking off from the habitual use of stimulating drinks:

℞.—Peppermint water, . . . . ℥ xij.
Sulphate of iron, . . . . . gr. v.
Spirits of nutmeg, . . . . ℥ ij.
Valirianate of quinia, . . . gr. ijss.

S. Teaspoonful taken as often as the desire for strong drink returns. I have had frequent occasion to test its efficacy in many cases in my practice, and have found it uniformly successful."

